# Sensing the Intentions to Speak in VR Group Discussions

**DOI:** 10.3390/s24020362

**Published:** 2024-01-07

**Authors:** Jiadong Chen, Chenghao Gu, Jiayi Zhang, Zhankun Liu, Shin‘ichi Konomi

**Affiliations:** 1 Graduate School of Information Science and Electrical Engineering, Kyushu University, Fukuoka 819-0395, Japan; chen.jiadong.450@s.kyushu-u.ac.jp (J.C.); gu.chenghao.564@s.kyushu-u.ac.jp (C.G.); zhang.jiayi.734@s.kyushu-u.ac.jp (J.Z.); liu.zhankun.907@s.kyushu-u.ac.jp (Z.L.); 2 Faculty of Arts and Science, Kyushu University, Fukuoka 819-0395, Japan

**Keywords:** virtual reality, human–computer interaction, sensor, deep learning, group work

## Abstract

While virtual reality (VR) technologies enable remote communication through the use of 3D avatars, it is often difficult to foster engaging group discussions without addressing the limitations to the non-verbal communication among distributed participants. In this paper, we discuss a technique to detect the intentions to speak in group discussions by tapping into intricate sensor data streams from VR headsets and hand-controllers. To this end, we developed a prototype VR group discussion app equipped with comprehensive sensor data-logging functions and conducted an experiment of VR group discussions (N = 24). We used the quantitative and qualitative experimental data to analyze participants’ experiences of group discussions in relation to the temporal patterns of their different speaking intentions. We then propose a sensor-based mechanism for detecting speaking intentions by employing a sampling strategy that considers the temporal patterns of speaking intentions, and we verify the feasibility of our approach in group discussion settings.

## 1. Introduction

The advancement of virtual reality (VR) technology has led to its widespread applications in various domains such as communication, tourism, education, and entertainment [1,2]. During the outbreak of COVID-19, VR has been explored as an alternative for conducting meetings when face-to-face communication was not possible due to lockdown measures. However, within the context of supporting these remote multi-user meetings, a persistent challenge has been the effective management of turn-taking in discussions [3,4]. The implementation of turn-taking in the conversation involves speakers and listeners closely observing each other to identify and receive signals for turn-taking [5]. However, the available features and range of social signals in virtual environments differ significantly from physical settings. Interaction in virtual environments often involves the use of avatars, which may have less expressive capabilities than our physical bodies. Additionally, the field of view in virtual environments may be narrower than that of humans, limiting our perception of the environment primarily through a low-bandwidth visual channel [6]. The limitations in expressive capacity and perception capabilities can have an impact on turn-taking in conversations, particularly when participants have to take the floor without being prompted by someone else. In this case, participants may find it challenging to capture the attention of others unless they are actively speaking or the current speaker has directed their focus toward them. Consequently, their social signals can be more challenging for others to notice. Therefore, our research will focus on situations within the turn transition where participants have to take the floor without being prompted by others, and we will refer to the intent to seize the floor as “speaking intention”, which is often included in social signals.

To the best of our knowledge, there has been no prior research addressing the concept of speaking intention among participants in VR group discussions. We believe that communication of speaking intention is of significant importance for managing participation in conversations and ensuring appropriate individual contribution opportunities in VR group discussions. In this paper, we present a research endeavor focused on investigating speaking intention, aiming to analyze the challenges associated with its presence in VR and explore the feasibility of detecting speaking intentions to assist group communication in VR environments. To detect speaking intentions, we primarily focus on sensor features available on VR devices including head-mounted displays and controllers. We draw upon prior research on non-verbal cues such as proximity cues and gaze in communication [7,8,9,10,11] and introduce relational features between two participants based on their individual sensor data. Firstly, we analyze real VR group discussions based on quantitative and qualitative data to uncover the patterns that inform the design of the detection mechanisms for speaking intentions. To do so, we recruited 24 participants for group discussions conducted in a VR environment. During the experiment, we collected sensor data and gathered speaking intention labels from participants through the cued retrospective approach. We next design the neural network-based approaches for detecting speaking intentions using the uncovered patterns, and we attained an accuracy of 62.79%. Our primary contributions are as follows:

1. We identified an asymmetry in how participants convey speaking intention in VR. They perceive that expressing their speaking intentions as relatively straightforward, but perceiving others’ speaking intentions is challenging.
2. We observed temporal patterns around speaking intentions as the intervals between the start of speaking intention and actual speaking are typically short, often lasting only around 1 s.
3. We show that our neural network-based approaches are effective in detecting speaking intentions by only using sensor data from off-the-shelf VR headsets and controllers. We also show that incorporating relational features between participants leads to minimal improvement in results.

## 2. Related Works

We employ VR for the support of social interactions, considering it not merely as a substitute for other social applications but as a medium that adds genuine value [12]. This is primarily attributed to its provision of enhanced expressiveness and a heightened sense of immersion from a technological standpoint [13]. VR has been demonstrated to provide support in general or intimate social scenarios [14], self-expression, and identity exploration [15], as well as skill and cultural learning [16,17,18]. However we also face challenges stemming from the absence of non-verbal cues. Tanenbaum et al., following a survey of mainstream social VR platforms, discuss the lack of support for two crucial social signals: facial expression control and unconscious body posture [19]. Bombari et al. also highlight non-verbal behavior as a significant design challenge in immersive interactive virtual environments [20]. In addressing this challenge, Lou et al. propose a solution employing additional electromyography (EMG) sensors to track facial muscle movements, using this information to reconstruct facial expressions for virtual avatars [21]. Kurzweg et al. found that important non-verbal communication cues, such as body language, were underrepresented in virtual meetings, resulting in a decline in the quality of communication. To remedy this, they suggest designing a series of body language to indicate participants’ conversation status, attention, and engagement, such as using gestures like drinking, typing, or answering a phone call to signify busyness [22]. In this paper, we also address the insufficient non-verbal cues on social interactions in VR particularly focusing on turn-taking in conversations, including situations where other participants are seeking opportunities to speak.

Turn-taking is an important part of any verbal interaction such as conversation, particularly in groups [23]. In group discussions, multiple participants come together and organize themselves for effective communication, assuming various roles such as speakers, addressees and side participants [24]. Within this dynamic, the turn-taking mechanism serves as a vital coordination tool, facilitating participants’ communicative actions to ensure smooth interactions among themselves. Therefore, researchers have explored the turn-taking mechanisms in communication from various perspectives. Jokinen et al. focused on identifying cues in human interaction that imply turn-taking. They highlighted the crucial role of gaze in coordinating turn-taking and the flow of conversational information, noting the significance of head movements in multiparty dialogues as well [11,25]. Streeck and Hartge discussed the role of gestures in turn-taking, observing that the gestures of listeners can serve as indicators of their desire to speak and as cues for the initiation of a new turn [26].

The relationship between these non-verbal cues and turn-taking lays the foundation for predicting turn-taking in conversations. Ishii et al. discussed a model for predicting the next speaker in multiparty meetings by focusing on the participants’ head movements [27]. Another model also developed by them predicts the next speaker based on non-verbal information in multiparty video conversation [28]. Furthermore, some researchers have focused on investigating turn-taking mechanisms in dialogues with the goal of improving human–machine interaction in conversational systems [29]. For instance, Ehret et al. enhanced embodied conversational agents (ECAs) by incorporating non-verbal features such as gestures and gaze to signal turn-taking, thereby making human–machine dialogues smoother and more enjoyable [30]. In the realm of voice-based human–machine interaction, managing turn-taking in conversations is a crucial area of focus [31,32]. Research in this field typically seeks to develop automated methods for predicting turn-taking based on conversational cues. When developing models to predict turn-taking, researchers often place a significant emphasis on syntax, semantics, pragmatics and prosody features [33,34,35]. However, in our research, the focus is on predicting the user’s speaking intentions. The focus is not solely on when turn-taking happens but also on identifying who triggers the turn-taking. For the participants initiating turn-taking, their verbal cues and prosody features are not available before they acquire the floor and begin speaking. Consequently, in our model development, we chose to concentrate on non-verbal cues recorded in sensor data.

Gibson et al. categorized turn transitions into four types (Turn Receiving: when a person speaks after he or she is addressed; Turn Claiming: when a person speaks after someone addressees the group as a whole; Turn Usurping: when a person speaks after someone else is addressed; Turn Continuing: when someone who is already in possession of the floor changes targets.) based on the participation framework: Turn Receiving, Turn Claiming, Turn Usurping and Turn Continuing [36,37]. In previous research, Turn Receiving in which the speaker relinquishes the floor and the addressee takes the turn, and Turn Continuing in which the speaker keeps the floor, have been extensively explored [38,39,40]. In our study, however, we will focus on situations where participants proactively take the speaking turn (i.e., Turn Claiming or Turn Usurping). We aim to leverage non-verbal cues from user behaviors recorded by sensors in VR devices to predict situations where individuals actively seek to speak during discussions. By doing so, we aim to facilitate a more seamless and engaging VR social interaction experience.

## 3. Experiment

**Figure 1:**
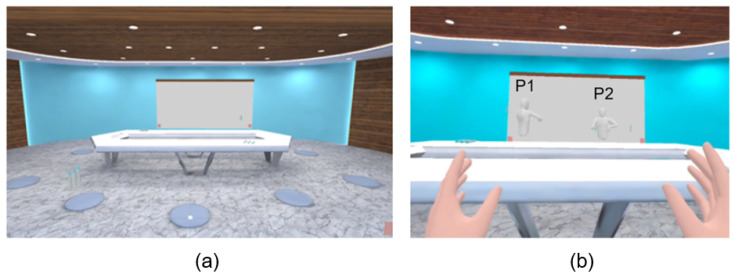
The virtual environment used in the experiment (**a**). Discussion taking place in the virtual environment (**b**).

We conducted a communication experiment in a VR environment with 24 participants, aiming to gain insights into participants’ speaking intentions in VR communication and to explore the feasibility of utilizing sensor data to detect speaking intentions. We envisioned a scenario of communication and discussion in a virtual space that is designed for a small number of participants. We organized participants into small groups of four, engaging them in a social game called “Two Truths and a Lie”. We employed the Oculus Quest 2 as the VR device for our experiment due to its affordability and widespread availability. This device includes two handheld controllers and a head-mounted display (HMD) and operates as an all-in-one VR system, allowing usage without being tethered to a computer. As the virtual environment for the experiment, we developed a VR multiplayer social application using Unity (Figure 1). In the virtual environment, we implemented a simplified avatar representation consisting of the head, upper torso, and hands. The application includes essential features for participant interaction, such as voice communication and a virtual whiteboard. Additionally, for data collection purposes, the application incorporates a data-recording function, enabling us to collect real-time sensor data from each participant’s VR device.

### 3.1. Data Collection

In the experiment, we collected and curated the data set comprising sensor data and labels indicating participants’ speaking intentions.

#### 3.1.1. Sensor Data

In the virtual environment, users can directly control their avatars through body movement. This is achieved by mapping users’ real-world movements onto the virtual environment using sensors on the VR device. Therefore, by recording sensor data, we can effectively reconstruct users’ behavior in the virtual environment.

Sensor data were automatically collected through our developed VR social application. During application usage, position, rotation, velocity, acceleration, angular velocity, and angular acceleration data were captured from three nodes (HMD, left hand controller and right hand controller) at a frequency of 20 Hz. Each type of data was represented in three dimensions corresponding to the X, Y, and Z axes. Consequently, every data frame obtained from the VR device consisted of 54 values (3 × 6 × 3). It is worth noting that within the virtual environment of the application, users can control the movement and rotation of their avatars in two ways: directly through body movements or by using the joystick on the hand controllers. Therefore, when considering the user’s position and orientation in the environment, we not only use data from sensors but also integrate the actions from the user’s controllers.

#### 3.1.2. Participant Annotated Labels

To obtain information from participants regarding their speaking intentions in communication, we employed a cued retrospective approach to collect these subjective data. Prior research has indicated the efficacy of retrospective methods with visual cues (such as images or videos) for accurate data collection in short-term studies [41,42]. Specifically, during the course of the experiment, we would capture first-person perspective videos for each participant within the virtual environment. After the experiment, participants were asked to review the recorded videos and identify instances of their intentions to take the floor actively and the moments when these intentions arised. We deliberately refrained from opting for an in-the-moment labeling approach during the experiment, which was primarily due to the concerns that it might influence participants’ natural speaking behavior.

**Figure 2:**
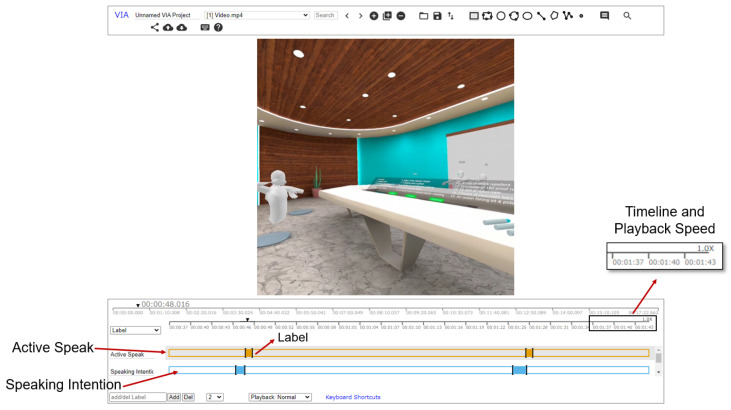
Interface of VGG Image Annotator. Participants annotate the time when speaking began in the active speak row. In the speaking intention line, the time when the intention to speak arose is annotated. The end time of the label does not require adjustment.

During the annotation process, we employed the VGG Image Annotator tool (Figure 2), which offers video annotation capabilities, allowing participants to add annotations on the timeline while reviewing video record. Participants have the flexibility to adjust the playback speed of the video, ranging from 0.1× to 16×. They can add annotations on the timeline and modify the start time and end time of labels. The minimum unit for label movement on the timeline is 20 ms. Using this tool, participants review the video and add labels to mark when they initiated taking the floor to speak or when their speaking intentions based on their recollection. Participants do not need to adjust the end time of the labels, simplifying the annotation task.

### 3.2. Two Truths and a Lie

Participants were divided into groups of four and engaged in a game of “Two Truths and a Lie” within the VR application we developed. In this game, group members took turns sharing three statements about themselves with one of the statements being false. After a participant’s presentation, the group members engaged in open discussions. For instance, they could ask the presenting participant for additional details to clarify statements or point out aspects they found suspicious. Following the discussions, the non-presenting participants were required to guess which statement was the lie. Once the presenter revealed the answer, the round continued to the next participant who then initiated their set of statements, and the process was repeated.

“Two Truths and a Lie” is a classic icebreaker activity commonly used to break the ice at social gatherings or group meetings. Such activities foster an energetic and positive discussion environment, allowing participants to relax and seamlessly integrate into the discussions [43,44]. The selection of this scenario was aimed at fostering speaking intentions among participants during the experiment. Throughout the game, participants had the freedom to move within the virtual environment as they like and were also able to utilize a virtual whiteboard. The game lasted approximately 15 min, during which researchers refrained from intervening. However, a timer was set to alert at 4-min intervals to prevent any individual participant’s turn from becoming excessively lengthy.

### 3.3. Participants

We recruited 24 participants from our university to take part in the experiment, consisting of 12 females and 12 males. They were university graduate students and one lecturer, aged 22–30 (M = 25.5, SD = 2.27). Among them, 13 individuals (54%) had prior experience with VR devices, while 11 (46%) had not used them before. Participants were randomly assigned to groups of four with the requirement that each group included two males and two females to maintain gender balance across the groups.

### 3.4. Experiment Procedure

Once a group of four participants arrived at the designated room, the formal experimental procedure commenced. Initially, participants were briefed on the process and purpose of the experiment along with the data collection requirements. Subsequently, we offered participants a guided tutorial on how to use the VR equipment and provided them with a comprehensive overview of the operational procedures of the application utilized during the experiment. Recognizing that some participants might not have prior experience with VR devices, approximately 30 min are allocated for participants to put on the HMD and familiarize themselves with the VR equipment to mitigate the novelty effect. During this period, participants who were new to VR were encouraged to engage with the built-in Oculus tutorial application The “First Step” was designed to facilitate the rapid comprehension of hand controller usage. Following the warm-up phase, participants entered the VR virtual meeting room to engage in a multiplayer interaction involving a social game of “Two Truths and a Lie” with a duration of approximately 15 min.

Throughout the experiment, participants remained seated while utilizing VR. The four participants were positioned in the corners of the room to ensure sufficient distance between them and prevent mutual interference. After the conclusion of the “Two Truths and One Lie” game, we introduced the annotation task to the participants. The instructions covered the definition of speech intentions and the usage of the annotation tools. Following the instructions, we provided each participant with a computer equipped with the annotation tool, and we allocated time for participants to practice using the annotation tools before commencing the formal annotation process, ensuring their proficiency in operating the annotation tools. The whole annotation process took approximately 30 min.

**Figure 3:**
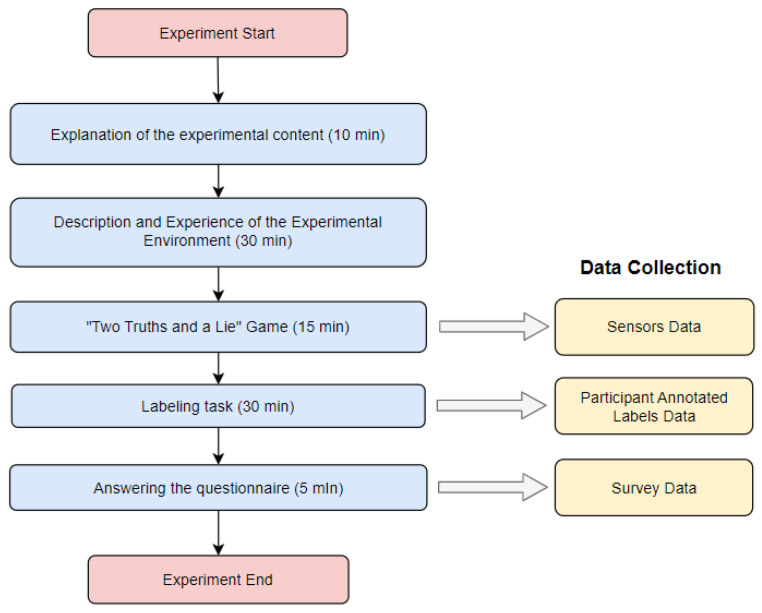
Flowchart of the experimental procedure.

Finally, each participant was required to complete a post-experiment questionnaire. The questionnaire encompassed participants’ experiences in conducting multiperson meetings in VR and their experiences related to speaking intentions during the experiment. The questionnaire included queries utilizing a 5-point Likert scale (1 = “Strongly Disagree” and 5 = “Strongly Agree”) and open-ended questions. Figure 3 depicts the experimental process with the data collected at each stage.

### 3.5. Data Processing

**Figure 4:**
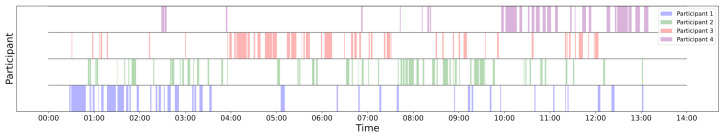
The segmentation results of utterance for a group (each row represents a participant). The colored sections indicate that the respective participant is speaking.

To capture participants’ complete conversational flow during the experiment, we conducted utterance segmentation on the collected video recordings. Drawing from prior research [45,46,47], we employed pauses as delimiters to segment utterance units. A pause exceeding 200 ms following a participant’s utterance was used as an utterance unit boundary. These utterance units were manually extracted by researchers based on audio cues. Figure 4 shows the utterance segmentation result for a group of four participants, and Table 1 presents the basic facts about utterance units. Subsequently, we corrected the speech start times annotated by participants using the results of utterance segmentation. The annotated start times by participants were replaced by the start times of the nearest utterance within a 1 s gap. This approach aims to minimize errors introduced by participant variations during timestamp annotations (such as some participants tending to annotate timestamps slightly slower or faster compared to the video).

In multimember conversations, features among members, such as spatial proximity to someone or gaze at someone, are significantly associated with turn-taking [8,9,39,48,49]. Therefore, in addition to using individual sensor data, we also computed and introduced relational features among members within the group. Based on the HMD positions, we computed the distances between participants and each of the other members as a feature representing proximity. Using HMD orientation, we computed the angle between the participant’s facial direction and the position of each other participants as a feature representing gaze. Additionally, recognizing the prominent role of the previous speaker during turn-taking, we introduced a speaking status feature for distinguishing the speaker within the group. The speaking status feature is a binary label that signifies whether each participant is speaking, which is determined by the results of utterance segmentation.

Consequently, we refer to the distance, angle, and speaking status features as relational features (with speaking status considered as a role-related relationship). Specifically, following the introduction of relational features, each data frame for participants is composed of a total of 63 numerical values derived from 54 + 3 × 3. Here, in “3 × 3”, the first “3” represents the other three individuals within the group, and the second “3” represents the three types of relational features.

## 4. Analysis of Experimental Data

In this section, we present the results of the analysis of the subjective data collected from participants along with the key insights we obtained regarding the speaking intention.

### 4.1. Questionnaire Result

**Figure 5:**
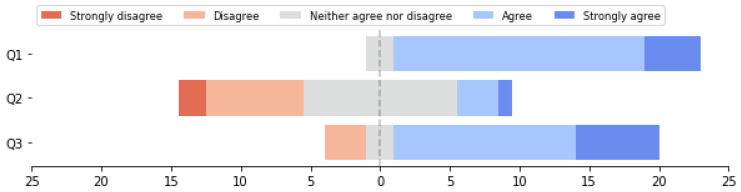
Participants’ responses to questions Q1–Q3. Horizontal axis is the number of participants.

At the end of the experiment, we conducted a post-experiment survey to inquire about participants’ perceptions of speaking intentions within the VR environment (see Table 2 and Figure 5). In the questions concerning the performance and perception of speaking intentions in the VR environment, participants generally found it easy to express their own speaking intention in the virtual environment (Mean = 4.08). However, discerning the speaking intention of others posed a challenge (Mean = 2.75). This outcome demonstrates the asymmetry in conveying and perceiving speaking intention when utilizing VR as a communication medium. Although VR provides participants with an environment highly resembling the real world, where users can directly control avatars through body movements, enabling them to express their intentions using non-verbal cues similar to face-to-face communication, technical and design-related issues hinder the perception of these cues by others. Regarding the influence of the ease of conveying and perceiving speaking intentions on group discussions, participants generally believed that reduced difficulty in conveying and perceiving speaking intentions was beneficial for productive group discussions (Mean = 3.91). Furthermore, we incorporated an open-ended question to investigate instances where participants had contemplated speaking during interactions but ultimately decided not to do so along with the reasons behind their decisions. Each response was coded by the researchers, and thematic patterns were subsequently extracted from these codes, as shown in Table 3. We found that the most significant cause of participants for abandoning their intention to speak is timing. After participants have expressed their intention to speak, if they are unable to gain the floor quickly, the topic will be pushed further by other participants. This can lead to the loss of currency in what the participant is trying to say and thus abandonment of the intention to speak.

### 4.2. Participant-Annotated Labeling Results

**Figure 6:**
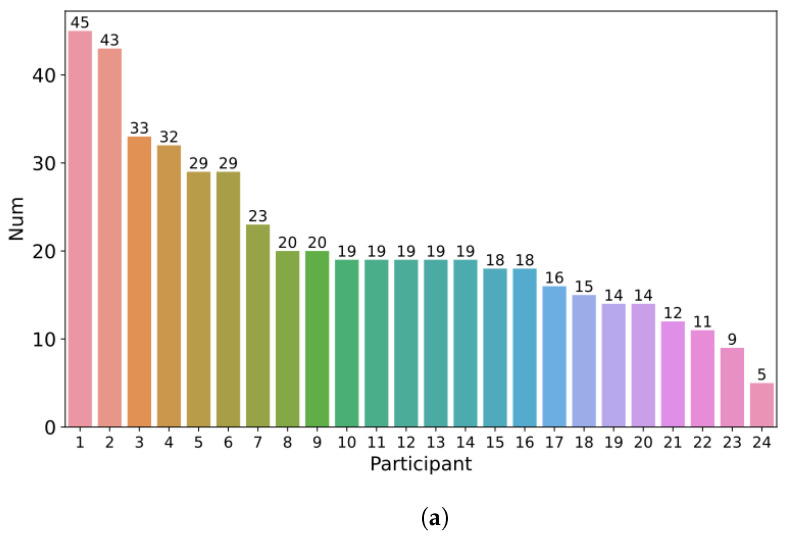

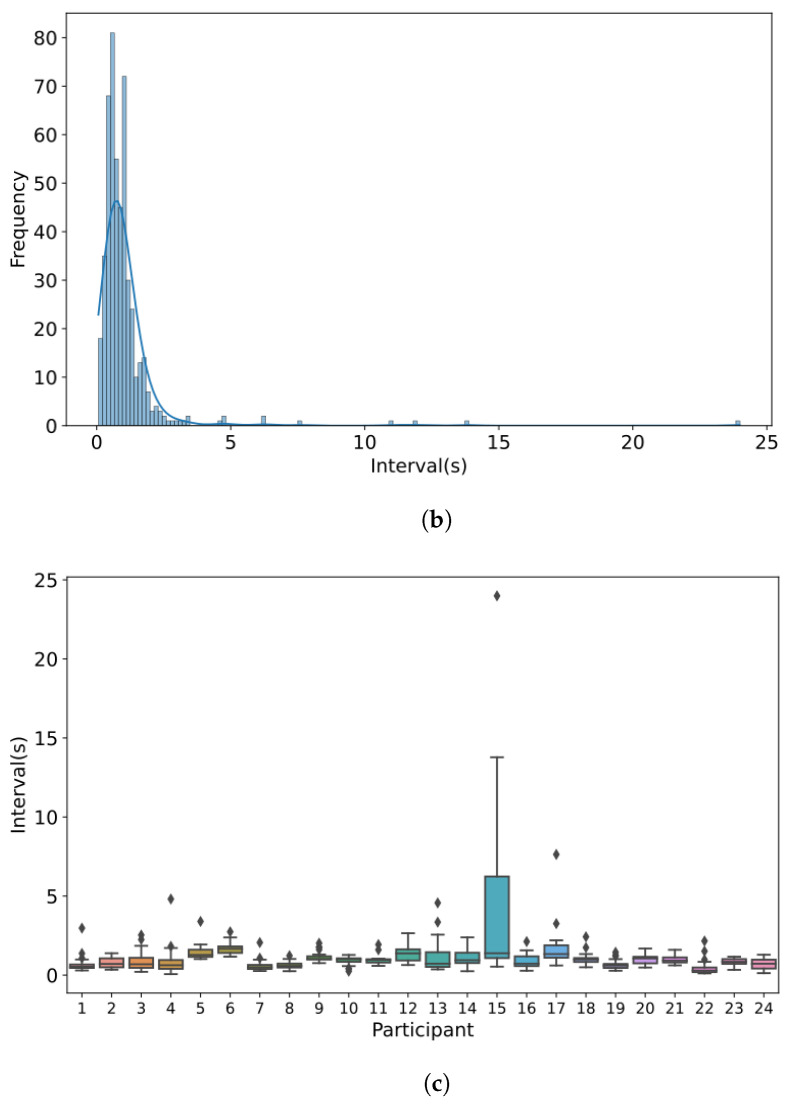
Analysis of participant-annotated labels. (**a**) Number of actively initiated speaking sequences; (**b**) distribution of intervals; (**c**) box plot of intervals. In Figures (**b**,**c**), the “interval” means the time gap between a participant forming the intent to speak and actually beginning to speak.

In the experiment, we collected a total of 501 active floor-taking labels from participants, which were paired with 501 corresponding speaking intention labels. Initially, we examined variations in the frequency of seizing the floor across different participants. As illustrated in Figure 6a, the highest number of frequency occurrences by a single participant was 45, while the lowest was 5 (Max: 45, Min: 5, Mean: 20.875, Std: 9.653). The results indicate the individual differences in the frequency of seizing the floor, which can be attributed to variations in participants’ personalities and other relevant traits. In order to explore the temporal patterns of speaking intention generation and the initiation of speech, we analyzed the time intervals between them. Figure 6b shows the distribution of time intervals, revealing that the intervals are primarily concentrated around 1 s (Max = 23.99, Min = 0.064, Mean = 1.055, Q1 = 0.518, Q2 = 0.792, Q3 = 1.111). This suggests that in most cases, participants execute their speech shortly after forming the speaking intention. Furthermore, we conducted an in-depth exploration into whether differences in time intervals existed across different participants. Our ANOVA result has shown significant discrepancies within the time interval data among the 24 participants (*p*-value: 2.23 × 10^−22^ < 0.01). To pinpoint these divergences, we performed multiple comparisons using Tukey’s honestly significant difference (HSD) method. The results indicate that the differences are only attributed to one participant who exhibits significant differences in comparison to all other participants (*p* = 0.05). However, there are no significant differences observed among the remaining participants. As shown in Figure 6c, participant 15 exhibits some notably long intervals, but the median interval time does not differ significantly from that of others. Upon reviewing video recordings of this participant, we found that the reason for these extended intervals is that other participants are firmly holding the floor when this participant forms the intention to speak, requiring him to wait for their speaking to conclude. This situation was also reported in the questionnaire, where other participants would abandon their own speaking intentions as a result. However, this participant did not easily abandon their speaking intentions when faced with the difficulty of obtaining the floor, instead opting to wait for an opportunity to express her opinions.

## 5. Detection of Speaking Intention

In this section, we examine the feasibility of detecting participants’ speaking intention based on the social signals embedded in sensor data. Employing a data-driven approach, we train the neural network model to perform the classification task between two categories: sensor data when participants exhibit speaking intention (positive class) and sensor data when participants do not exhibit speaking intention (negative class). In the following subsections, we first introduced our data sampling strategy. Subsequently, we utilized the data collected in the experiment to train and test three widely used time-series data classification models, presenting the results for each model. Additionally, for each model, we compared the impact of using different features on the model’s performance.

### 5.1. Data Sampling Strategy

For the purpose of speaking intention detection, our initial step involves filtering out sensor data corresponding to participants’ speech moments. Utilizing the results of our utterance segmentation, we obtain the speaking state of participants at each moment. In practical applications, this information can also be acquired through microphones. For all remaining sensor data points during non-speaking states, we select a sampling window of 3 s prior to the participant’s active initiation of speech. This selection is based on the temporal patterns associated with speaking intention.

Specifically, within this window, we designate the 1.5 s period immediately preceding the onset of speech as the sampling region for positive samples. This decision is supported by the fact that this interval can encompass the vast majority of instances indicative of speaking intention as illustrated in Figure 6b, where 1.5 s > 1.1 s, which is the third quartile. Conversely, the interval between 1.5 and 3 s prior to the start of speech is designated as the sampling region for negative samples. This approach offers two key advantages. Firstly, it allows for a balanced size of positive and negative samples. Secondly, it reduces interference from unrelated behaviors. Throughout the entire communication session, participants spend an average of 715.1 s in a non-speaking state in contrast to an average of only 22 s when participants exhibit speaking intention. Furthermore, during non-speaking states, participants are likely to disengage from the communication process. For example, we observed that some participants engage in activities such as drawing on the whiteboard or exploring the virtual environment while others are engaged in communication. These behaviors fall outside the scope of communication and introduce noise into the detection process. Therefore, we consider sampling in the proximity of the time point when participants initiate speech to avoid capturing data during the time when participants have disengaged from the communication context. Additionally, referring to the statistics of the time intervals between two consecutive utterances by participants (Table 1, with a median of 4.62 s), the chosen 3 s window aligns well with the typical intervals between participant speech during regular communication.

**Figure 7:**
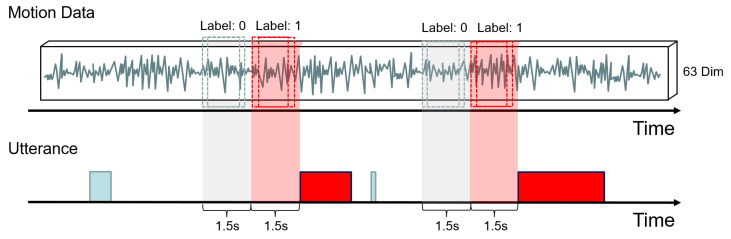
Sampling process. The motion data represent the data obtained from the sensor of the participant’s VR device. These data along with the relational features result in 63 dimensions. The utterance indicates the participant’s utterance units, where red indicates the unit labeled by the participant as actively speaking. In the light red region of length 1.5 s, we sample positive samples. In the light gray region of 1.5 s, we sample negative samples.

In the sampling regions for positive and negative samples, we employed a sliding window approach to extract sensor data with a window size of 25 and a step size of 1. Figure 7 illustrates the sampling process. In total, we collected 2447 samples, comprising 1303 positive samples and 1144 negative samples.

### 5.2. Neural Network Model

Due to the success of neural network (NN)-based methods in various tasks involving time-series classification, such as anomaly detection [50], human activity recognition [51], and gaze pattern recognition [52], we have chosen an NN-based approach to process the time-series sensor data. Specifically, we input the sampled data along with their corresponding labels into a time-series neural network model. The network autonomously extracts features from the data and performs binary classification through a fully connected layer with a sigmoid activation function. Regarding the NN architectures employed for handling time-series data, we experimented with several commonly used time-series classification architectures, which included the following: EEG Net [53], an architecture primarily composed of two convolutional steps, first the temporal convolution and then the depthwise convolution, MLSTM-FCN [54], an architecture that combines both one-dimensional convolutional neural networks (1D-CNNs) and long short-term memory (LSTM) layers, and InceptionTime [55], an architecture inspired by Google’s Inception network [56], which is also based on convolution layers.

The specific architecture details of the model can be found in Appendix A (Table A1 and Table A2).

### 5.3. Model Performance

During model performance validation, we used widely adopted metrics, including accuracy, precision, recall, and F1 score, which are common for evaluating classification model performance. Additionally, we calculated the area under receiver operating characteristic (AUROC), which is a metric that evaluates the model’s overall discriminating ability between positive and negative samples across different thresholds.

**Figure 8:**
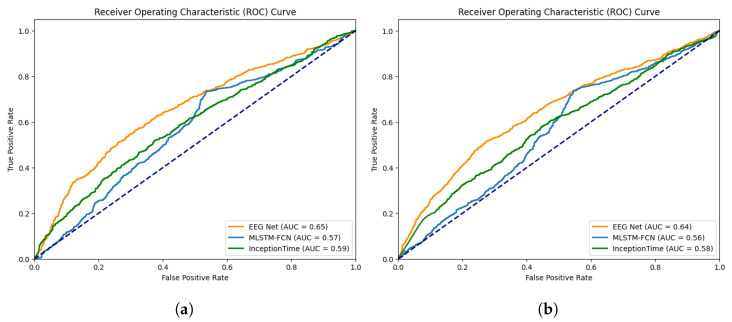
ROC curves of models (**a**) Sensor data + relational features; (**b**) Only sensor data. “AUC”: AUROC.

To assess the generalization performance of features across participants, we employed leave-one-out cross-validation. Specifically, during each model training iteration, we selected one participant’s data as the validation set while using the data from the remaining participants as the training set. Since we had a total of 24 participants, this model training process was repeated 24 times. After completing the training for all models, we calculated the average performance metrics as the measure of model performance. Table 4 and Figure 8 show the performance metrics and ROC curves for each neural network architecture. We introduced random prediction as a baseline to assess whether sensor data contribute to speaking intention recognition. This baseline model randomly assigns samples to positive or negative classes with a 50% probability.

Overall, EEG Net achieved the highest accuracy (0.6279) and precision (0.6738). MLSTM-FCN attained the highest recall (0.7352) and F1 score (0.6881). However, InceptionTime did not achieve the best performance in any of the metrics. Next, when observing the receiver operating characteristic (ROC) curves, EEG Net exhibited the best discriminating ability between positive and negative samples with an AUROC of 0.65.

Furthermore, we examine the impact of the introduced relational features in the detection task. However, directly calculating the importance of features in neural networks is not straightforward. Therefore, we attempted to compare the model’s performance with and without the inclusion of relational features, measuring feature importance based on the performance difference. This approach is frequently used when examining specific features or modules within neural networks [57,58]. Table 4 (Only Sensor Data) shows the performance metrics of models that do not utilize relational features. The results indicate that models without relational features generally exhibit slightly weaker performance compared to models with these features. However, the recall (0.6312) for EEG Net and the recall (0.5966) and F1 score (0.5919) for InceptionTime improved slightly compared to the same architectures with relational features. Nevertheless, none of them reached the best performance. When looking at the ROC curves, models without relational features demonstrated slightly inferior performance compared to those using relational features. However, overall, the difference in performance between models with and without relational features was minimal, suggesting that the impact of relational features on speaking intention detection is limited.

## 6. Discussion

### 6.1. Speaking Intention in VR

Through the annotations provided by participants, we investigated the temporal patterns of participants in generating the intention to speak and taking the floor. The results indicate that in the majority of the cases, the interval between the generation of speaking intentions by participants and the commencement of speaking was mostly around 1 s with only very few instances exceeding 5 s. In our experiment, these longer intervals were primarily associated with a participant who appeared to be more ‘patient’ compared to others. However, the vast majority of participants did not display such patience. Their speaking intentions were typically generated shortly before speaking. Those participants who could not gain the floor within a short timeframe to express their opinions often abandoned their intention to speak. This is also corroborated by our questionnaire analysis, as most participants reported timing as the primary reason for abandoning their speaking intentions. Furthermore, these results also imply that the inability to perform effective turn-taking regulation in a conversation can lead to missed opportunities for acquiring opinions. Additionally, through the questionnaire, we also investigated participants’ perceptions of conveying speaking intentions in the VR environment. Participants found it easy to express their speaking intentions in VR, but perceiving the speaking intentions of others was challenging. This asymmetry could lead to situations where participants believe they have expressed their speaking intentions, but others have not noticed. If a participant starts speaking directly in such situations, it is unpredictable for other participants. This can lead to confusion in turn management and increase the likelihood of dialogue overlap. Similar findings have been reported in previous research on web conferences [59,60], where verbal conflicts occurred more frequently than in face-to-face situations.

### 6.2. Assistance Based on Speaking Intention Detection

For the challenges related to speaking intent in VR, we will discuss the possibilities of providing assistance to VR discussion based on participants’ speaking intention detection from both real-time and non-real-time perspectives.

#### 6.2.1. Real Time

Multiplayer interactions conducted using VR represent a form of technologically mediated communication that allows designers to strategically separate non-verbal signals transmitted by one interactant from those received by others [61]. Non-verbal signals can be enhanced or attenuated through carefully designed virtual environments, influencing the interactions among participants. For example, some research has artificially presented non-verbal cues in VR environments and explored their impact on communication [62,63]. Similarly, when it comes to participants’ speaking intentions, we can consider designing a presentation method to enhance them. Enhanced speech intentions can be made more noticeable to other participants, addressing the issue of perceptibility caused by the low fidelity of VR. With such assistance, participants can better coordinate their conversational exchanges in communication, thereby improving the efficiency of group interactions. Participants in our survey also agreed that being able to perceive others’ speaking intentions easily contributes to communication in VR.

#### 6.2.2. Non-Real Time

In scenarios where group work or collaboration occurs in VR, tracking the frequency of participants expressing speaking intentions can serve as a metric for analyzing or improving interactions among participants. We think that speaking intentions provide a novel perspective for assessing engagement in communication. While this is somewhat similar to the use of total speaking time [64,65] or frequency of turn-taking [66], which have been applied in previous research, speaking intentions arguably reflect participants’ proactivity and their interest in the discussion content more accurately during the conversation. By combining the analysis of speaking intentions with other metrics, we can gain deeper insights into group interactions. For example, if a participant has many speaking intentions but speaks infrequently, it may indicate that they are facing some obstacles to expressing their opinions. Conversely, if someone has few speaking intentions but speaks frequently, it could suggest that they are being forced to speak by others in the communication. By adjusting the factors that influence interaction, we can improve the balance of the conversation, thereby enhancing the performance and creativity in group work [67,68].

### 6.3. Speaking Intention Detection Based on Sensor Data

We classified the sensor data of participants before and after they had speaking intention to examine whether speaking intention detection could be achieved by capturing social signals from sensor data. The results indicate that the models based on neural networks achieved an accuracy of 0.6279, a precision of 0.6738, a recall of 0.7352, an F1 score of 0.6881, and an AUROC of 0.65. Specifically, EEG Net achieved the best accurary, precision and AUROC, while MLSTM-FCN attained the best recall and F1 score. In practical applications, model selection may depend on the specific context. For instance, when providing real-time feedback on speaking intention, precision becomes crucial, as false positive feedback on speaking intention can disrupt communication. However, for statistical speaking intention analysis during the communication process, recall might be of higher importance.

Additionally, we introduced relational features among participants and tested their importance in speech intent detection. The results revealed that models using relational features showed slight performance improvements, but the improvements were limited (an increase of 0.0121 in the best F1 score). This suggests that relational features did not play a significant role in speaking intention detection.

### 6.4. Limitation and Future Work

Our experiments were conducted in a laboratory environment; therefore, some of the experimental conditions inevitably influenced the participants’ communication behavior. For instance, participants reported in the questionnaires that their reluctance to express intentions to speak was due to the added workload of labeling as well as the time constraints during the experiment. Since speaking intentions are subjective and challenging to observe, we could not eliminate the step of participant annotation. However, considering simplifying the task or employing additional assistive tools may help alleviate participants’ concerns about the workload.

In this study, based on our sampling method, we tested the feasibility of using data from embedded sensors in VR devices to detect speaking intentions only within a 3 s interval before participants started speaking. This still presents a gap in applying speaking intention detection in a wider range of practical scenarios. Ideally, the model should be capable of detecting speaking intentions in any segment of data sampled from participants’ communication. This is an exceptionally challenging task, as it implies that the model must distinguish between behaviors when participants have the intention to speak and all other potential behaviors during communication. Therefore, the primary focus of future work will be to explore speaking intention detection methods that can be applied to a wider range of scenarios. We will attempt to detect speaking intentions within a broader sampling range and consider integrating additional contextual information to eliminate situations where detection or assistance is unnecessary, thus mitigating the challenges posed by the participants’ diverse behaviors during communication.

## 7. Conclusions

In the VR environment, the low fidelity in replicating the physical world leads to the deficiency of non-verbal cues, thereby posing challenges for user interactions. To address this challenge, we aimed to provide assistance to participants in VR by using sensor data from VR devices. In this study, we focused on turn-taking in group communication and explored the difficulties encountered by participants in expressing speaking intentions and acquiring the right to speak. We conducted a small-group communication experiment in VR, during which we collected and built a dataset consisting of sensor data and speaking intention labels.

We identified asymmetry in the transmission of speaking intentions in the VR environment through questionnaires. Analysis of the labels provided by participants yielded significant insights into speaking intentions. Building on these insights, we explored the feasibility of using sensor data to detect speaking intentions. Our comparison of the three neural network-based models indicated that the models can distinguish participants’ motion data based on the presence or absence of speaking intentions, outperforming random classification across various evaluation metrics. However, surprisingly, the introduced relational features among participants had a very limited impact on detection improvement. We also discussed the potential for using speaking intention detection to assist interactions in the VR environment. We believe that our work represents a first significant step toward providing assistance in small group interactions in VR from the perspective of speaking intentions.

## Figures and Tables

**Table 1:** Through video recording, we have divided a total of 1441 utterance units. “Duration” represents the duration of each utterance unit in seconds. “Interval” represents the time interval between two consecutive utterance units from the same person in seconds.

Statistics	Mean	SD	Median	Max	Min
Duration	3.08	4.47	1.82	72.03	0.17
Interval	13.06	24.02	4.62	229.15	0.24

**Table 2:** Post-questionnaire questions.

	Question	Type
Q1	Do you think it is easy to express your speaking intentions in the virtual environment?	5-Point Likert Scale
Q2	Do you think it is easy to perceive the speaking intentions of others in the virtual environment?	5-Point Likert Scale
Q3	Do you think that perceiving and expressing speaking intentions more easily would be beneficial for group discussions?	5-Point Likert Scale
Q4	Have you ever had situations during the discussion where you wanted to say something but finally didn’t? If yes, please write down the reason.	Open-Ended Question

**Table 3:** The coding result for Q4: Why did you give up your intention to speak?

Theme	Code	Count
Timing Reasons	Difficulty interrupting others	4
	Taking too long to organize their thoughts	3
	Topic has changed	2
Content Reasons	The content is irrelevant to the current topic	4
	Someone else already mentioned the same thing	3
Social Etiquette	Worried about offending others	2
	Worried about talking too much	1
Experimental Setup	Don’t want to increase the workload of labeling	2
	Experiment time is limited	1
None	None (No instance of giving up)	5

**Table 4:** Model performance with relational features and without relational features. “Acc.”: Accuracy, “Prec.”: Precision, “F1.”: F1 score.

	Sensor Data + Relational Features				Only Sensor Data			
Metrics	Acc.	Prec.	Recall	F1	Acc.	Prec.	Recall	F1
Baseline	0.4879	0.5221	0.4956	0.5085	0.4879	0.5221	0.4956	0.5085
EEG-Net	**0.6279**	**0.6738**	0.6099	0.6403	0.6164	0.6156	0.6312	0.6233
MLSTM-FCN	0.6207	0.6466	0.7352	**0.6881**	0.6115	**0.6261**	**0.7345**	**0.6760**
InceptionTime	0.5654	0.6058	0.5621	0.5831	0.5653	0.5872	0.5966	0.5919

## Data Availability

The data presented in this study are available upon request from the corresponding author. The data are not publicly available due to privacy reasons.

## Appendix A. Detail of the Models

In Table A1 and Table A2, we list the detailed structure of the models used, including the configuration of each layer, the size of the kernel k, the probability of dropout layer p, the hidden size h of the LSTM, the reduction r of the Squeeze-and-Excitation block (SE-Block) [69], and the output dimension $d_{\mathrm{out}\;}$ of the fully connected layer.

**Table A1:** Layer details of our EGG Net and MLSTM-FCN model. C is the number of channels in time-series data. Legend: “Conv1D”: 1D Convolution Layer, “BN”: Batch Normalization, “AvgPool”: Average Pooling Layer, “DimShuffle”: Dimension Shuffle, “DepConv”: Depthwise Convolution Layer, “SepConv”: Separable Convolution Layer [53].

(a) EGG Net		
**EEG Net Layer**		**Input Shape**
Conv2D	k=(1,10)	C×25
BN		8×C×25
DepConv	k=(C,1)	8×C×25
BN + ELU		16×1×25
AvgPooling	k=(1,2)	16×1×25
Dropout	p=0.25	16×1×12
SepConv	k=(1,16)	16×1×12
BN + ELU		16×1×12
AvgPooling	k=(1,4)	16×1×12
Dropout	p=0.25	16×1×3
Flatten		16×1×3
Dense	$d_{\mathrm{out}\;}=1$	48
(b) MLSTM-FCN		
**MLSTM-FCN Layer**		**Input Shape**
Conv1D	k=8	C×25
BN + ReLU		128×25
SE-Block	r=16	128×25
Conv1D	k=5	128×25
BN + ReLU		256×25
SE-Block	r=16	256×25
Conv1D	k=3	256×25
BN + ReLU		128×25
GlobalAvgPool		128×25
DimShuffle		C×25
LSTM	h=8	25×C
Dropout	p=0.25	8
Concate		128, 8
Dense	$d_{\mathrm{out}\;}=1$	136

**Table A2:** Layer details of our InceptionTime model. The structure in the first block is the “Inception Module”. The “pd” in the Maxpooling layer indicates that padding should be used to keep the output size equal to the input.

Inception Time Layer		Input Shape	Connected to
Conv1D	k=1	C×25	InputLayer
MaxPooling1D	k=3, pd	C×25	InputLayer
Conv1D_1_	k=20	32×25	Conv1D
Conv1D_2_	k=10	32×25	Conv1D
Conv1D_3_	k=5	32×25	Conv1D
Conv1D_4_	k=1	C×25	MaxPooling1D
Concate		4×32×25	Conv1D_1_,Conv1D_2_
			Conv1D_3_,Conv1D_4_
BN		128×25	Concate
ReLU		128×25	BN
Inception Module			
BN		128×25	Concate
ReLU		128×25	BN
Inception Module			
Conv1D	k=1	C×25	InputLayer
$BN_{1}$		128×25	Concate
$BN_{2}$		128×25	Conv1D
ReLU		128×25	$BN_{1}$
Add		128×25	$BN_{1}$ , $BN_{2}$
$ReLU_{1}$		128×25	Add
Inception Module			
BN		128×25	Concate
ReLU		128×25	BN
Inception Module			
BN		128×25	Concate
ReLU		128×25	BN
Inception Module			
Conv1D	k=1	128×25	$ReLU_{1}$
$BN_{1}$		128×25	Concate
$BN_{2}$		128×25	Conv1D
ReLU		128×25	$BN_{1}$
Add		128×25	ReLU, $BN_{2}$
ReLU		128×25	Add
GlobalAvgPool1D		128×25	
Dense	$d_{\mathrm{out}\;}=1$	128	
